# Twenty Years’ Experience in Retroperitoneal Lymph Node Dissection for Testicular Cancer in a Tertiary Referral Center

**DOI:** 10.3390/medicina59010133

**Published:** 2023-01-10

**Authors:** Angelo Mottaran, Amelio Ercolino, Lorenzo Bianchi, Pietro Piazza, Francesco Manes, Sasan Amirhassankhani, Marco Salvador, Francesco Chessa, Beniamino Corcioni, Alessandro Bertaccini, Riccardo Schiavina, Eugenio Brunocilla

**Affiliations:** 1 Division of Urology, IRCCS Azienda Ospedaliero—Universitaria di Bologna, 40138 Bologna, Italy; 2 Department of Experimental, Diagnostic and Specialty Medicine (DIMES), School of Medicine, University of Bologna, 40126 Bologna, Italy; 3 Department of Radiology, IRCCS Azienda Ospedaliero—Universitaria di Bologna, 40138 Bologna, Italy

**Keywords:** retroperitoneal lymph node dissection, open approach, testicular cancer, primary setting, secondary setting, RPLND, surgical volume

## Abstract

*Background and Objectives*: The aim of this article is to present a single-surgeon, open retroperitoneal lymph node dissection (RPLND) series for testicular cancer in a high-volume center. *Materials and Methods*: We reviewed data from patients who underwent RPLND performed by an experienced surgeon at our institution between 2000 and 2019. We evaluated surgical and perioperative outcomes, complications, Recurrence-Free Survival (RFS), Overall Survival (OS), and Cancer-Specific Survival (CSS). *Results*: RPLND was performed in primary and secondary settings in 21 (32%) and 44 (68%) patients, respectively. Median operative time was 180 min. Median hospital stay was 6 days. Complications occurred in 23 (35%) patients, with 9 (14%) events reported as Clavien grade ≥ 3. Patients in the primary RPLND group were significantly younger, more likely to have NSGCT, had higher clinical N0 and M0, and had higher nerve-sparing RPLND (all *p* ≤ 0.04) compared to those in the secondary RPLND group. In the median follow-up of 120 (56–180) months, 10 (15%) patients experienced recurrence. Finally, 20-year OS, CSS, and RFS were 89%, 92%, and 85%, respectively, with no significant difference in survival rates between primary vs. secondary RPLND subgroups (*p* = 0.64, *p* = 0.7, and *p* = 0.31, respectively). *Conclusions*: Open RPLND performed by an experienced high-volume surgeon achieves excellent oncological and functional outcomes supporting the centralization of these complex procedures.

## 1. Introduction

Testicular cancer (TC) represents the most common solid tumor among males between 15 and 40 years old [1]. The incidence of testicular cancer has increased over the past several decades for unknown reasons [2,3]. TC is a very aggressive disease, with up to 20% of patients at risk of developing a retroperitoneal mass [4]. Over the last decade, however, cure rates have increased thanks to the availability of multiple and effective adjuvant treatment options. Treatments after orchiectomy range from active surveillance to retroperitoneal lymph node dissection (RPLND), chemotherapy, and radiotherapy, depending on different histological factors and stage of the disease [5]. RPLND is a key component of the multimodal treatment, and considering the high cure rates achieved by surgery, its role and indications have evolved in both low-stage and advanced testicular cancer [4]. The American Urological Association guidelines recommend RPLND both as the primary treatment and if chemotherapy has failed to eradicate the residual retroperitoneal disease [6]. The European Association of Urology guidelines recommend RPLND in case of failed chemotherapy, when a teratoma is found on orchiectomy pathology, and in select patients with high-risk oncological features or poor compliance with follow-up [7]. However, RPLND is a challenging surgical procedure affected by a huge incidence of intraoperative and postoperative complications. The incidence of overall postoperative complications is up to 25% and up to 35% in a primary vs. secondary setting, respectively. The main complications include vascular injuries, anejaculation, venous thromboembolism, pulmonary embolism, chylous ascites, lymphocele, neuropraxia, small bowel obstruction, ileus, infections, and acute respiratory distress syndrome.

For a long time, open RPLND (O-RPLND) has been considered the standard of care and still represents the benchmark for the comparison of oncological and functional outcomes with other surgical techniques, especially considering the lack of data on long-term oncological outcomes for robotic RPLND and the inherent difficulties associated with laparoscopic RPLND [8,9,10,11,12,13].

Many studies have focused on the role of the center surgical volume and the surgeon’s experience [14,15,16]. Complication rates directly correlate with surgeon volume and experience; therefore, NICE and the European Consensus Conference on Diagnosis and Treatment of Germ Cell Cancer recommended performing RPLND only in specialized centers, regardless of the surgical approach [17,18].

The current study aims to present our single-surgeon, open RPLND series in a high-volume center within a period of 20 years.

## 2. Materials and Methods

### 2.1. Study Design and Population

The current study relied on a prospectively maintained institutional reviewed board-approved database (IRB approval 3237). It included patients who underwent RPLND between 2000 and 2019 in a single high-volume center (IRCCS Azienda Ospedaliero-Universitaria di Bologna, Policlinico Sant’Orsola-Malpighi, Bologna, Italy).

**Figure 1:**
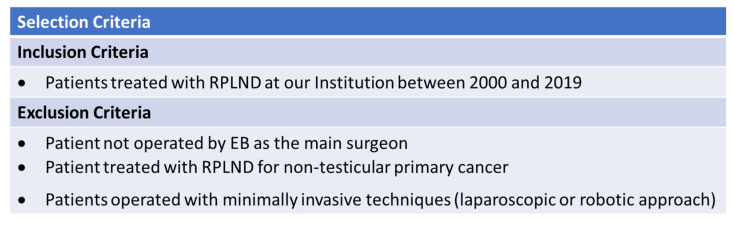
Inclusion and exclusion criteria.

Demographic characteristics at baseline, perioperative and pathological data, complications, and surgical and oncological outcomes were collected for all patients. All surgeries included in our analysis were performed by a single experienced open surgeon (EB). Of 85 patients operated on by EB as the main surgeon, 20 people were treated with a minimally invasive technique (laparoscopic or robotic approach) or referred to RPLND for non-testicular primary cancer and, therefore, excluded from the analysis. The inclusion and exclusion criteria are summarized in Figure 1. This yielded a final population of 65 patients. This study was conducted in accordance with good clinical practice guidelines and the ethical principles of the Declaration of Helsinki.

### 2.2. Outcomes

The primary outcome was to evaluate oncological outcomes, namely Overall Survival (OS) and Recurrence-Free Survival (RFS), of O-RPLND. The secondary outcome was to describe intraoperative and postoperative complications, including ejaculatory disorders. A retrospective collection of overall early (≤90-days) postoperative complications was performed based on patient charts and follow-up interviews by two medical doctors (FM and MS) to perform an accurate evaluation of adverse events. Postoperative complications were defined according to the Common Terminology Criteria for Adverse Events (CTCAE) version 5.0 [19] and graded according to the Clavien–Dindo classification [20]. The reports of complications followed the quality criteria for accurate and comprehensive reporting of surgical outcomes recommended by the EAU Guidelines [21].

### 2.3. Surgical Technique and Surgeon Experience

The surgical approach was formulated via shared decision-making by the patient and the surgeon, with considerations for histologic findings, staging, adjacent organ involvement, and treatment effects, as demonstrated on preoperative imaging. The patient was placed in the supine position, then an open surgical approach was performed. After a median xifo-pubic incision, a transperitoneal approach was applied, mobilizing the bowel, exposing the retroperitoneum, and split-and-rolling the nodal tissue off the major vessels with a unilateral or bilateral template, according to the preoperative imaging. Unilateral template dissection was performed only in patients with low-volume metastatic disease limited to the primary landing zone of the affected testis. The right-sided template included the right common iliac and the paracaval, precaval, retrocaval, and interaortocaval lymph nodes. The left-sided template included the left common iliac and the pre-aortic, para-aortic, and retro-aortic lymph nodes to the level of the inferior mesenteric artery.

At the beginning of the study period, the main surgeon (EB) had already completed over 3000 open major surgeries as the first surgeon, including O-RPNLDs, radical prostatectomies, radical cystectomies with intracorporeal and extracorporeal urinary diversions, partial and radical nephrectomies, nephroureterectomies, pyeloplasties, and complex pelvic surgeries. Therefore, the surgical learning curve was not evaluated in this study.

### 2.4. Statistical Analysis

For patients who fulfill the selection criteria, the following data were collected:

- Preoperative information (risk factors, date of birth, date at surgery, TNM-staging, Union for International Cancer Control staging, preoperative testis histology, post-chemotherapy mass size and site, chemotherapy regimens, indication for RPLND, radiology reports, hematic exams, and tumor markers).
- Perioperative information and surgical outcomes (date of surgery, location, operation report, nerve-sparing approach, template, concurrent procedures, necessity of transfusion, and intraoperative complications).
- Postoperative information (complications, length of stay, resected mass pathologic report, radiology reports, hematic exams, follow-up, recurrence, survival). Pathologic review included nodal yield, histology of the retroperitoneal mass excised, number of positive lymph nodes, and final pathologic nodal staging.
- Follow-up information (complications, ejaculation preservation and, recurrence). Complications were identified by reviewing operative records, and postoperative in- and outpatient records. Complications were graded according to the Clavien–Dindo Classification. Relapse and survival data were obtained from medical records and external correspondence. Computed tomography (CT) scans or magnetic resonance imaging (MRI) as well as tumor serum markers were used to evaluate disease recurrence.

Statistical analyses, reporting, and interpretation of the results were conducted according to established guidelines [22].

Firstly, we assessed the study population’s preoperative clinical and pathological characteristics. Secondly, we evaluated surgical features, perioperative outcomes, and complications. We also compared perioperative characteristics between different subgroups of patients: patients with seminomatous germ cell tumors (SGCT) vs. NSGCT and patients who underwent RPLND in a primary vs. secondary setting. Surgery in chemo-naive patients with high-risk stage I disease or stage II disease with negative serum markers was defined as primary RPLND.

Surgery in post-chemotherapy patients with residual mass and negative serum markers was defined as secondary RPLND.

Descriptive statistics included frequencies and proportions for categorical variables. Medians with interquartile ranges (IQR) were reported for continuously coded variables.

Chi-square and Kruskal–Wallis tests were performed to compare medians and proportions of continuous variables among the groups regarding baseline and perioperative characteristics, as well as oncological outcomes among groups.

Kaplan–Meier survival analysis with the log-rank test was performed to assess the Recurrence-Free Survival [RFS] and Overall Survival [OS] of the overall population after stratification according to the RPLND setting. All statistical tests were performed using IBM SPSS (Statistics for Windows, Version 23.0), with a 2-sided significance level set at *p* < 0.05.

## 3. Results

### 3.1. Patients’ Characteristics

Table 1 depicts characteristics of the overall population after orchiectomy and prior to RPLND. Overall, 21 (32%) and 44 (68%) patients underwent RPLND in a primary and secondary setting, respectively. The median age was 30 (IQR 2742) years.

**Figure 2:**
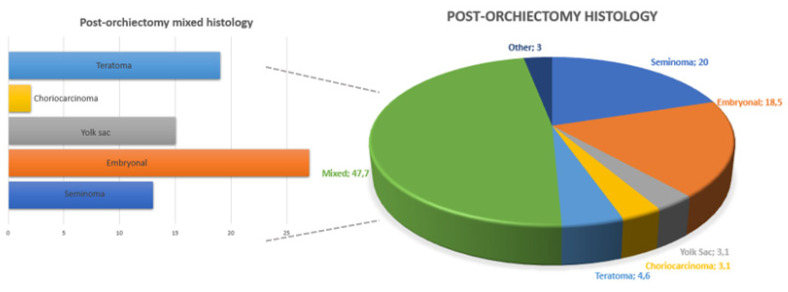
Overall and mixed distribution of testicular cancer histotypes at post-orchiectomy pathology.

Figure 2 shows the distribution of different histology after the primary orchiectomy. In 13 patients (20%), the primary tumor was a pure seminoma, whereas 52 patients (80%) had an NSGCT.

Moreover, 39 patients (60%) had normal tumor markers at diagnosis (S0 stage), whereas 26 (40%) patients had S ≥ 1 stage. The Union for International Cancer Control (UICC) stage was I, II, and III in 21 (32%), 28 (44%), and 16 (24%) patients, respectively. After the orchiectomy, 7 patients (11%) were referred to surveillance, 43 (66%) underwent first-line chemotherapy, and 16 patients (25%) received additional adjuvant treatments after one course of first-line therapy.

**Figure 3:**
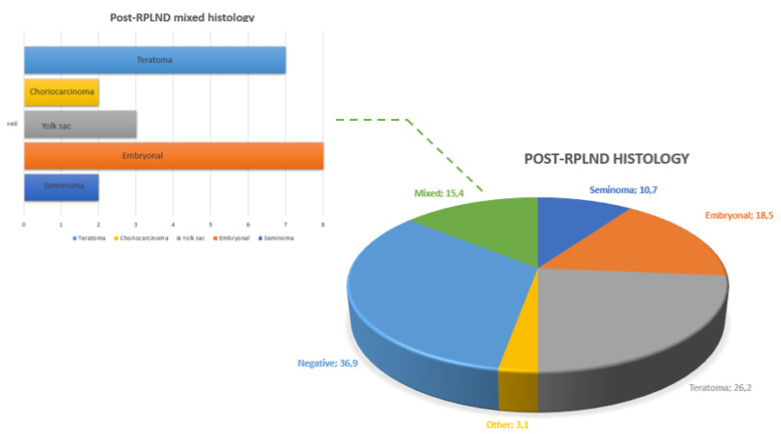
Overall and mixed distribution of testicular cancer histotypes at post-RPLND pathology.

Post-chemotherapy nodal residual masses (PCM) were found in 42 (65%) patients. Of these, nine (14%) patients also had a visceral mass. The median size of the PCM was 34 mm (IQR 20-49). Figure 3 summarizes the distribution of different histotypes after RPLND.

### 3.2. Clinical and Pathological Features among Groups according to Primary Histology or RPLND Setting

Patients with NSGCT were significantly younger (29 vs. 39 years, *p* = 0.04), presented with higher S stage (45% vs. 28%, *p* = 0.03), were more likely to undergo adjuvant chemotherapy (23% vs. 7%, *p* = 0.02), and had a significant lower lymph node recurrence rate (89% vs. 69%, *p* = 0.03) compared with the seminoma group (Table 2).

Table 3 shows baseline characteristics according to lymph node dissection setting (primary vs. secondary RPLND). Patients undergoing primary RPLND compared to secondary RPLND were significantly younger (27 vs. 33 years, *p* = 0.02), were more likely to have a NSGCT (90% vs. 64%, *p* = 0.02) at primary histology, had higher N0 (57% vs. 30%, *p* = 0.04) and M0 stage rates (100% vs. 75%, *p* = 0.04), and were more likely to receive adjuvant chemotherapy (33% vs. 16%, *p* = 0.003). Patients undergoing secondary RPLND had a higher clinical stage (IIC-III, 45% vs. 5%, *p* = 0.03) and were included in a poorer prognostic group (39% vs. 5%, *p* = 0.01). If a median (IQR) follow-up of 120 (56–180) months is considered, 10 patients (15%) had a post-RPLND relapse at a median (IQR) time of 10 (8–24) months from surgery.

### 3.3. Perioperative Characteristics, Complications, and Functional Outcomes

**Figure 4:**
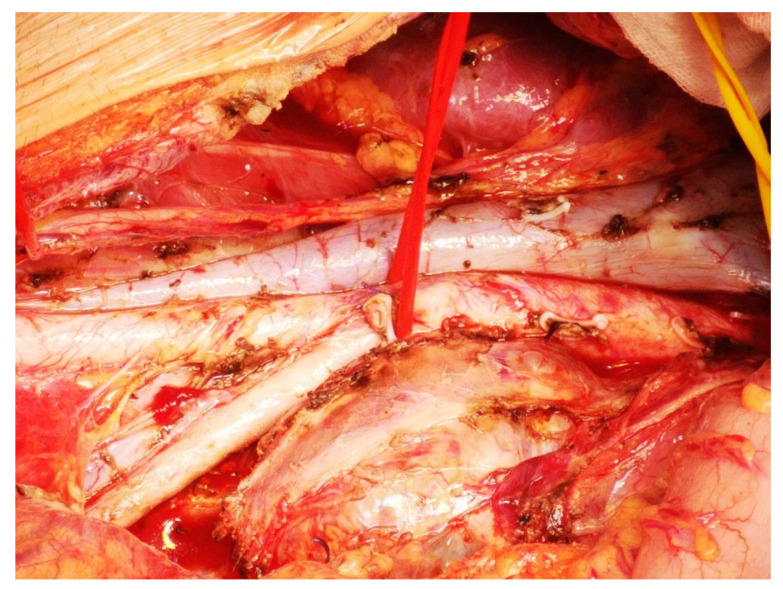
Bilateral RPLND template with inferior mesenteric artery dissection.

**Figure 5:**
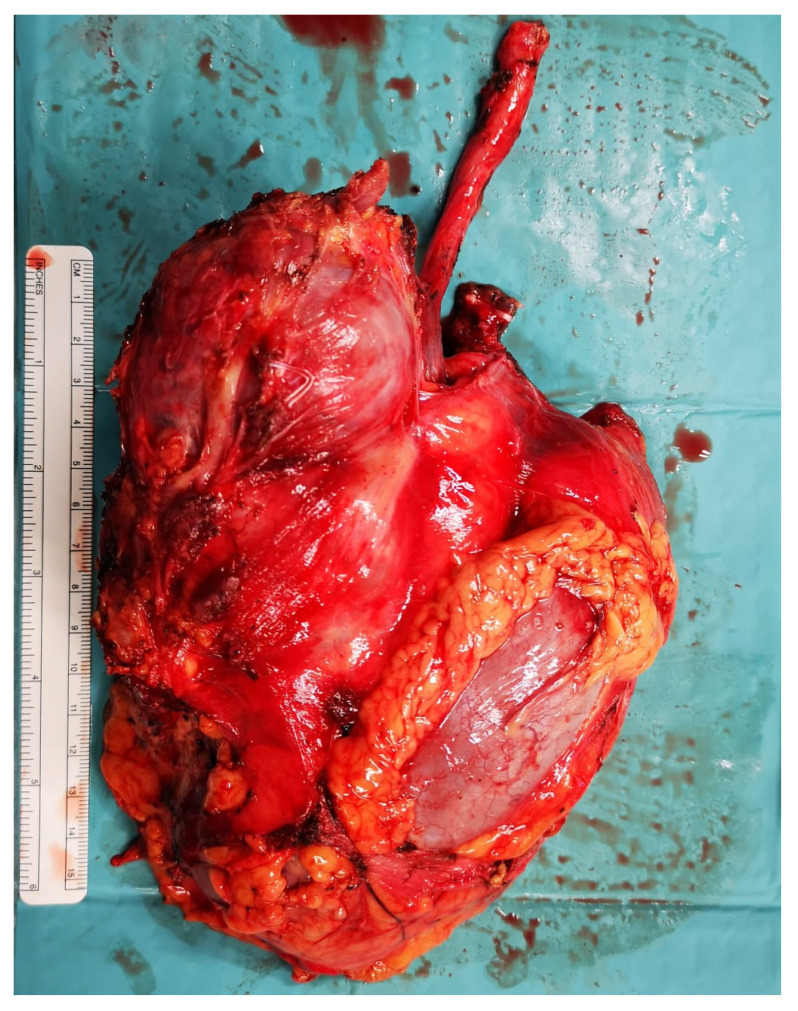
Radical nephrectomy during retroperitoneal lymph node dissection.

A unilateral dissection template was performed in 33 patients (51%), whereas 32 cases (49%) underwent a bilateral template (Figure 4). A nerve-sparing approach was performed in 23 patients (35%) among the bilateral template group. Additional surgical procedures were required in 12 (18.5%) patients. Radical nephrectomy (Figure 5) was performed as an additional procedure in six (9.2%) patients, followed by inferior mesenteric artery resection in three (4.6%) patients, adrenalectomy in two (3.1%) patients, and ureteral resection in one (1.5%) patient.

The median operative time was 180 min (IQR 150-233). The median estimated blood loss was 150 mL (IQR 80-170), with two patients (3%) requiring intraoperative transfusion and seven patients (11%) requiring postoperative transfusion.

Intraoperative complications occurred in four patients (6%), all consisting of vascular lesions. Postoperative complications occurred in 23 cases (35%). The median postoperative length of stay was 6 days (IQR 5-8), and in five cases (8%), readmission was required.

Pathological examination revealed no residual vital cancer in 24 cases (37%). Teratoma was the most represented histotype (42%), followed by embryonic carcinoma (18.5%) and mixed tumor (15.4%).

### 3.4. Oncological Outcomes

**Figure 6:**
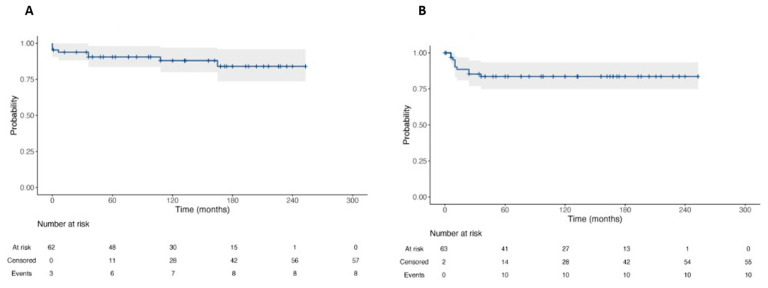
Kaplan–Meier Curve for Overall Survival (**A**) and Recurrence-Free Survival (**B**) of patients with testicular cancer and submitted to RPLND.

**Figure 7:**
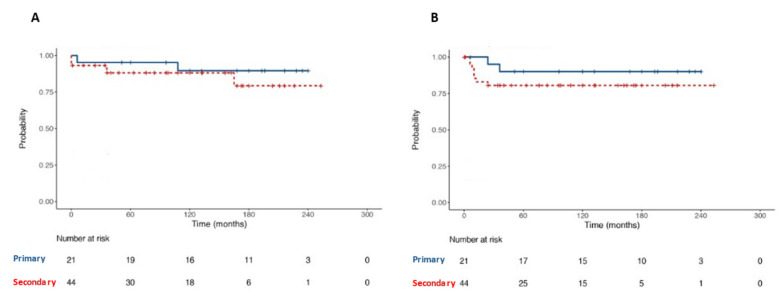
Kaplan–Meier with log-rank test for comparison of Overall Survival (**A**) and Recurrence-Free Survival (**B**) between primary and secondary RPLND subgroups patients.

At survival analyses, OS and RFS were 87.7% and 84.6% at 20-year follow-up, respectively (Figure 6). No statistically significant difference was found in terms of OS among patients undergoing primary RPLND vs. secondary RPLND (*p* = 0.44). Likewise, no statistically significant difference was found in terms of RFS among patients undergoing primary RPLND vs. secondary RPLND (*p* = 0.31, Figure 7).

## 4. Discussion

RPLND in the primary setting has been used recently for stage I and IIA high-risk SGCTs with negative serum markers. RPLND in the secondary setting is traditionally used for the treatment of residual masses larger than 3 cm for SGCTs and larger than 1 cm for metastatic NCGCTs with normal tumor markers [7]. The role of RPLND in sub-centimeter residual masses remains controversial, given that 30% of sub-centimeter masses have been found to be teratoma or viable non-teratomatous germ cell tumors [7].

We aimed to present the characteristics and long-term oncological and functional outcomes of a large cohort of patients undergoing O-RPLND for testicular cancer. Our analyses showed several noteworthy observations.

First, in our population, we observed a strong prevalence of embryonal carcinoma, teratoma, and yolk sac tumor among the post-orchiectomy histotypes. Final pathology revealed 26% of pure teratoma prevalence other than the presence of a teratomatous component in all the mixed tumors of our series. Despite the high morbidity rates associated with RPLND, the use of surgery as a primary treatment avoids the toxicity associated with chemotherapy and allows the resection of teratomatous masses liable for malignant progression and transformation. The comparison between groups of patients according to the RPLND setting revealed that patients undergoing primary RPLND have more frequently a stage I or a stage IIA indeed.

Second, surgical expertise, technical skill, and an adequate center volume are required in order to achieve optimal outcomes for patients undergoing RPLND. The benefits of surgical experience have been highlighted in a United States study by Tandstad et al., which found surgical quality to be essential to ensuring optimal perioperative, functional, and oncological outcomes [23]. Moreover, data from the United States Surveillance, Epidemiology and End Results (SEER) database showed significantly increased perioperative morbidity and mortality in patients treated in “non-referral” centers [16]. Following the Improving Outcomes Guidance in the UK in 2002, specialist testicular cancer services were established in the UK to perform all RPLND, utilizing specialized high-volume surgeons only. Following this centralization in the UK, Wells et al. prospectively examined RPLND surgery for testicular cancer over 1 year. This study found the mean range of RPLND per center was 9 (2–32) with a reported minimum of six cases per surgeon in order to lower the rates of perioperative morbidity [24]. In Italy, there is no requirement that RPLND only be performed in specialist centers by high-volume surgeons. Our center is a referral oncological center, the largest in the Emilia-Romagna region, with an average of eight RPLND per year. The reported perioperative outcomes are in line with data from studies conducted in other referral centers, confirming the importance of surgical volume.

Third, a major factor adding to the RPLND complexity arises in cases in which local structures are invaded, necessitating nephrectomy, vascular dissection, or repair of vascular structures such as the aorta and vena cava. In our cohort, 18.5% of patients required additional surgery. This data is in line with the available literature, which reports up to 27% of additional surgery rate in a secondary setting.

The median value of blood loss and the operative time were 150 mL and 180 min, respectively. These data are strongly better than the available literature, emphasizing again the importance of surgical expertise. The operative time is particularly important considering that the literature demonstrated significantly higher complication rates in patients with a duration of surgery exceeding 270 min [25]. The length of stay was 6 (IQR 5-8) days in line with the best largest series in the UK.

The total postoperative complication rate in our series was 35%, whereas high-grade complications (Clavien ≥ 3) occurred in 14% of patients perfectly in line with a recent meta-analysis and the range described in the literature between 12% and 19% [25,26]. The literature has proven that the rates of intra- and postoperative complication, as well as those of additional surgical procedures, are higher in patients with an intermediate or poor prognostic group and in patients with teratomatous or seminomatous components. In our cohort, 35% of patients have an intermediate or poor prognostic group. The prevalent histotype is teratoma, whereas seminoma accounts for 10% of pure tumors after RPLND and is the third most frequent component in mixed ones. Furthermore, intraoperative complications in our cohort resulted to be 6%, in line with previously published data following primary RPLND and half of the average rate found in the literature after post-chemotherapy RPLND.

Fourth, in our cohort, 35% of the entire population underwent a nerve-sparing dissection. Patients referred to primary RPLND more frequently had a nerve-sparing dissection compared to those undergoing PC-RPLND (57% vs. 25%, *p* = 0.01) due to the high prevalence of poor prognostic group patients. These data are in line with the current indications of performing a nerve-sparing dissection in a secondary setting only in patients with a post-chemotherapy mass size less than 5 cm and with an intermediate–good prognostic group. In our cohort, ejaculation was preserved in 85% of patients, proving the quality of RPLND performed with the maintenance of ejaculation even at very long follow-up.

Fifth, survival analyses showed a two-phased pattern in OS, with the first decrease after 10 months corresponding to an increased disease recurrence, and the second fall at 30 months in the context of late relapses that arise after a median time of at least 2 years. Such relapses are usually related to poor prognosis in affected patients. This pattern is strictly related to chemotherapy late toxicity, considering also that 18% of patients in our cohort underwent a second line of therapy. The prognostic group has been considered the most important prognostic factor and independent predictor of relapse. This consideration explains the relapse rate at 15% of our population, which includes 1/3 of patients in the intermediate–poor prognostic group. Of note, no differences were found in OS and RFS between patients undergoing primary vs. secondary RPLND. This shows that an appropriate setting and multimodal approach in a high-volume center guarantees excellent results from the oncological point of view and ensures that these can be maintained over time.

Despite its strengths, our study is not devoid of limitations. Firstly, the study relied on a retrospective series, with all its inherent limitations. Secondly, we could not adjust for surgical equipment modification over time. Thirdly, we reported data from a highly experienced surgeon with a high annual caseload and extensive open and robotic experience. Therefore, these outcomes may not be generalizable to other centers.

However, to the best of our knowledge, we report oncological outcomes of RPLND in a high-volume center and with an experienced surgeon with the longest follow-up available in the literature to date. Finally, the reliability of our data collection is high because all the quality criteria for accurate and comprehensive reporting of surgical outcomes recommended by EAU Guidelines on reporting and grading of complications were fulfilled (14/14 criteria satisfied). This is crucial to increasing the rate of complications detected, as recently demonstrated [27].

Finally, we think that retroperitoneal lymph node dissection for testicular cancer should be centralized in a few high-volume specialized centers. Each center should have a multi-specialist team including a urologist, a radiologist, a radiation oncologist, and an oncologist. Furthermore, each center should be the reference point for a population of no less than 3–4 million inhabitants. Once this type of model has been implemented in many countries, national and international prospective multicenter databases could provide valuable oncological and functional data on lymphadenectomies performed in primary and secondary settings in high-volume surgical centers.

## 5. Conclusions

This high-volume single-surgeon case series analysis showed excellent oncologic and functional outcomes of RPLND performed for testicular cancer in different settings over 10 years of follow-up. The perioperative outcomes, complications, and oncologic results are comparable with those found in high-volume international centers of excellence that perform RPLND. This study supports that the centralization of these complex procedures to high-volume surgeons in high-volume centers improves functional and oncological outcomes.

## Figures and Tables

**Table 1:** Clinical and pathological characteristics of the study population after orchiectomy and prior RPLND.

Characteristics	Overall (n = 65)
Age (years)	
Median (IQR)	30 (27–42)
Clinical primary tumor side, n (%)	
Right	35 (54)
Left	30 (46)
Pre-orchiectomy markers value, median (IQR)	
AFP (ng/mL)	17 (3–247)
Beta-HCG (×10^3^ mIU/mL)	21 (5–59)
LDH (U/L)	238 (151–855)
Pathologic T stage, n (%)	
pTis-pT1	24 (37)
pT2	29 (45)
p ≥ T3	12 (19)
Post-orchiectomy markers value, median (IQR)	
AFP (ng/mL)	18 (4–445)
Beta-HCG (×10^3^ mIU/mL)	7.0 (2–162)
LDH (U/L)	157 (124–238)
Clinical nodal status, n (%)	
cN0	25 (38)
cN1	14 (22)
cN2	17 (26)
cN3	9 (14)
Clinical M stage, n (%)	
cM0	54 (83)
cM1a	8 (12)
cM1b	3 (5)
UICC Stage, n (%)	
IA, IB	17 (26)
IS	4 (6)
IIA	12 (19)
IIB	13 (20)
IIC	3 (5)
III	16 (24)
PCM, n (%)	
Overall	42 (65)
Nodal	42 (65)
Visceral	9 (14)
PCM diameter (mm)	
Median (IQR)	34 (20–49)

IQR—interquartile ranges; AFP—alpha-fetoprotein; UICC—Union for International Cancer Control; PCM—post-chemotherapy residual mass.

**Table 2:** Clinical and pathological characteristics of the study population among seminomatous and non-seminomatous groups prior to RPLND.

	Overall	SGCT Group	NSGCT Group	*p* Value
Number of patients, n (%)	65	18 (28)	47 (72)	-
Age				
Median (IQR)	30 (27–42)	39 (30–50)	29 (26–39)	0.04
S Stage, n (%)				
S0	39 (60)	13 (72)	26 (55)	0.03
S ≥ 1	26 (40)	5 (28)	21 (45)	
Adjuvant CT, n (%)	14 (22)	3 (17)	11 (23)	0.02
Recurrence after orchiectomy, n (%)				
Overall	47 (72.3)	16 (89)	31 (66)	0.06
Nodal	43 (67.8)	16 (89)	28 (60)	0.03
Visceral	5 (7.6)	3 (17)	2 (4)	0.06
Time to recurrence (months)				
Median (IQR)	6 (3–22)	6 (4–10)	12 (10–24)	0.07

IQR: interquartile ranges; CT: Chemotherapy.

**Table 3:** Clinical and pathological characteristics of the study population among primary and secondary RPLND groups.

	Overall	Primary RPLND	Secondary RPLND	*p* Value
Number of patients, n (%)	65	21 (32)	44 (68)	-
Age				
Median (IQR)	30 (27–42)	27 (24–35)	33 (27–44)	0.02
Primary histology, n (%)				
Seminoma	27 (42)	9 (43)	18 (41)	
Embryonal	41 (63)	19 (90)	22 (50)	
Teratoma	21 (32)	7 (33)	14 (32)	0.09
Yolk sac	17 (26)	9 (43)	8 (18)	
Choriocarcinoma	5 (8)	1 (5)	4 (9)	
Mixed	30 (46)	14 (67)	16 (36)	
Other	2 (5)	-	2 (5)	
Primary histological group, n (%)				
Seminomatous	18 (28)	2 (10)	16 (36)	0.02
Non-seminomatous	47 (72)	19 (90)	28 (64)	
Post-orchiectomy AFP				
Median (IQR)	18.0 (4–445)	4.0 (2.5–6.8)	123 (8–742)	0.02
Clinical nodal status, n (%)				
cN0	25 (38)	12 (57)	13 (30)	
cN1	14 (22)	5 (24)	9 (20)	0.04
cN2	17 (26)	4 (19)	13 (30)	
cN3	9 (14)	-	9 (20)	
Clinical M stage, n (%)				
cM0	54 (83)	21 (100)	33 (75)	
cM1a	8 (12)	-	8 (18)	0.04
cM1b	3 (5)	-	3 (7)	
UICC Stage, n (%)				
IA,IB	17 (26)	8 (38)	9 (20)	
IS	4 (6)	4 (19)	-	
IIA	12 (19)	4 (19)	8 (18)	0.03
IIB	13 (20)	4 (19)	9 (20)	
IIC	3 (5)	-	3 (7)	
III	16 (24)	1 (5)	15 (35)	
Surveillance post orchiectomy, n (%)	7 (11)	5 (24)	2 (5)	0.02
Adjuvant CT post orchiectomy, n (%)	14 (22)	7 (33)	7 (16)	0.003
*Nerve-sparing* RPLND, n (%)	23 (35)	12 (57)	11 (25)	0.01
Further surgical procedures during RPLND, n(%)				
Overall	42 (65)	16 (76)	26 (59)	0.18
Operative time (min)				
Median (IQR)	180 (150–233)	175 (125–217)	180 (158–260)	0.17
Estimated Blood Loss (EBL), mL				
Median (IQR)	150 (80–170)	130 (84–183)	150 (80–205)	0.96
Transfusions, n (%)				
Intraoperative	2 (3)	-	2 (5)	0.32
Postoperative	7 (11)	1 (5)	6 (14)	0.28
Nodal Yield (n)				
Median (IQR)	16 (11–23)	16 (12–26)	15 (11–21)	0.75
Positive nodes (n)				
Median (IQR)	1 (0–2)	0 (0–1)	1 (0–2)	0.03
Nonvital cancer, n (%)	25 (39)	14 (67)	11 (25)	<0.001
Intraoperative complications, n (%)	4 (6)	0 (0)	4 (9)	0.15
Postoperative complications, n (%)	23 (35)	8 (38)	15 (34)	0.60
Postoperative complications, n (%)				
CD ≤ 2	14 (22)	8 (38)	6 (14)	0.12
CD ≥ 3	9 (14)	-	9 (20)	
Length of stay (LOS), days				
Median (IQR)	6 (5–8)	6 (5–7)	7 (5–9)	0.07
Readmissions, n (%)	5 (8)	1 (5)	4 (9)	0.54
Anejaculation, n (%)	10 (15)	1 (5)	9 (21)	0.10
Recurrence after RPLND, n (%)				
Overall	10 (15)	2 (10)	8 (18)	0.37
Time to recurrence (months)				
Median (IQR)	10 (8–24)	30 (24–46)	10 (7–12)	0.04
Overall survival, % (n)	57 (88)	19 (90.5)	38 (86)	0.64
Cancer-specific survival, % (n)	60 (92)	19 (90.5)	41 (93)	0.70

IQR—interquartile ranges; RPLND—retroperitoneal lymph node dissection; AFP—alpha-fetoprotein; CT—chemotherapy; LOS—length of stay; UICC—Union for International Cancer Control.

## Data Availability

Data are collected in a prospectively maintained institutional reviewed board-approved database.
